# Neuronal Spike Initiation Modulated by Extracellular Electric Fields

**DOI:** 10.1371/journal.pone.0097481

**Published:** 2014-05-29

**Authors:** Guo-Sheng Yi, Jiang Wang, Xi-Le Wei, Kai-Ming Tsang, Wai-Lok Chan, Bin Deng

**Affiliations:** 1 School of Electrical Engineering and Automation, Tianjin University, Tianjin, China; 2 Department of Electrical Engineering, The Hong Kong Polytechnic University, Hong Kong, China; Dalhousie University, Canada

## Abstract

Based on a reduced two-compartment model, the dynamical and biophysical mechanism underlying the spike initiation of the neuron to extracellular electric fields is investigated in this paper. With stability and phase plane analysis, we first investigate in detail the dynamical properties of neuronal spike initiation induced by geometric parameter and internal coupling conductance. The geometric parameter is the ratio between soma area and total membrane area, which describes the proportion of area occupied by somatic chamber. It is found that varying it could qualitatively alter the bifurcation structures of equilibrium as well as neuronal phase portraits, which remain unchanged when varying internal coupling conductance. By analyzing the activating properties of somatic membrane currents at subthreshold potentials, we explore the relevant biophysical basis of spike initiation dynamics induced by these two parameters. It is observed that increasing geometric parameter could greatly decrease the intensity of the internal current flowing from soma to dendrite, which switches spike initiation dynamics from Hopf bifurcation to SNIC bifurcation; increasing internal coupling conductance could lead to the increase of this outward internal current, whereas the increasing range is so small that it could not qualitatively alter the spike initiation dynamics. These results highlight that neuronal geometric parameter is a crucial factor in determining the spike initiation dynamics to electric fields. The finding is useful to interpret the functional significance of neuronal biophysical properties in their encoding dynamics, which could contribute to uncovering how neuron encodes electric field signals.

## Introduction

Electromagnetic field stimulation, as a noninvasive brain modulation technique, nowadays has been successfully and widely used in the clinic to treat and study various neurologic, psychiatric and pain disorders [1]–[3]. The commonly used techniques encompass transcranial magnetic stimulation (TMS), repetitive TMS (rTMS), electroconvulsive therapy (ECT), transcranial direct current stimulation (tDCS), low field magnetic stimulation (LFMS), and so on [1], [2], [4]–[7]. This special stimulus modality could induce an electric field in the extracellular space around the interested brain tissue to modulate relevant neuronal activity and ultimate behavior [3], [8]. One fundamental question of this technique needed to be solved is that the precise mechanism underlying neuronal excitation by electromagnetic stimulus is still unclear [1]–[3], [8], [9]. Addressing this question requires the knowledge of how the induced extracellular electric field interacts with neuronal encoding dynamics [3], [8].

There have been many experimental studies regarding the interactions between electric field and neuronal encoding dynamics. It has been shown that extracellular electric field is capable of modulating the excitability of many neurons [10]–[13]. For example, it could suppress [14], [15] and entrain epileptiform activities [16], promote burst firing [9], affect cortical wave propagation [17], and alter action potential threshold or timing [9], [18]. Moreover, unlike invasive current stimulus, the electric field stimulus could induce spatial polarization in neurons [9]–[11], [19], [20]. The neuronal segments near the stimulating anode are hyperpolarized, and simultaneously the segments near cathode are depolarized. These neuromodulatory effects of electric field are governed by both of electric field characteristics and neuronal physiological properties, especially morphological features [9], [19]–[22]. However, the relevant mechanisms underlying these effects are still unclear.

In fact, how neuron encodes stimulus information has been shown to derive from their spike initiation dynamics [23], [24]. It mainly refers to how different membrane variables interact at the subthreshold potentials, which could decipher neuron adopts what rules to determine when and why they spike, i.e., how an individual spike is initiated. In computational neuroscience, the dynamical mechanisms of spike initiation are usually studied with nonlinear dynamical system theory, such as phase plane, stability or bifurcation analysis [23]–[28]. With these methods, the underlying mechanisms of abundant neuronal properties to external current stimulus are uncovered, such as adaptation [29], [30], sensitivity [31], Hodgkin's three classes of excitability [24], bursting [26], synchronization [26], [27], and so on. Moreover, neuronal encoding schemes to external information are also tightly related to the properties of vast ionic channels in neuron membranes, and many researches adopt it to interpret the biophysical basis of spike initiation. For example, Prescott et al found that the relative activation properties between inward and outward ionic currents could lead to different bifurcation mechanism of spike initiation [24]; Wester and Contreras shown that Na^+^ channel inactivation and K^+^ channel activation could both control the relationship between spike threshold and the rate of rise of the membrane potential, which plays a crucial role in spike initiation [32]. Thus, translating dynamical explanations of spike initiation into biophysically interpretation could provide greater insight into neural encoding [24]. However, there are still no studies using these methods to investigate the spike initiation dynamics of the neuron to electric fields.

In our previous study [33], we first proposed a reduced two-compartment neuron model to explore how extracellular electric field modulates neuron activity. The model not only can represent the main biophysical characteristics of field effects, i.e., spatial polarization, but also incorporates a geometric parameter which describes the proportion of area occupied by soma. Both of them are crucial factors in determining neuronal response to electric field stimulus [8]–[11], [19]–[22], [34]. What is more, it is very suitable for explaining the dynamical mechanism and relevant biophysical basis for electric field effects. Depending on geometric parameter and internal coupling conductance between two compartments, we have extensively studied the spiking behavior and bifurcation structure of the model to extracellular electric fields. It has been found that varying geometric parameter could switch neuronal bifurcation from Hopf to SNIC, while varying internal coupling conductance could only make spike generation through SNIC bifurcation. Further, we have also shown that the electric field threshold for triggering neuron spiking is determined by geometric parameter, which is consistent with previous experimental [9], [21], [22] and modeling [8] predictions. However, there is still lack of a complete description of the dynamical mechanisms and especially biophysical basis of neuronal spike initiation to electric fields, which leaves some important questions unanswered. How do two parameters induce different effects on the bifurcation structure of the neuron to electric fields? What biophysical properties are qualitatively affected by geometric parameter to alter the bifurcation structure, and why varying internal coupling conductance could not?

To solve these problems, the study first adopts phase plane and stability analysis to further investigate the dynamical properties of the spike initiation with different geometric and internal coupling parameters. Then, we explore their relevant biophysical basis by analyzing the activation properties of different somatic membrane currents at the subthreshold potentials. All these mechanistic investigations of spike initiation could help us uncover how neuron encodes electric field stimulus.

## Model

**Figure 1:**
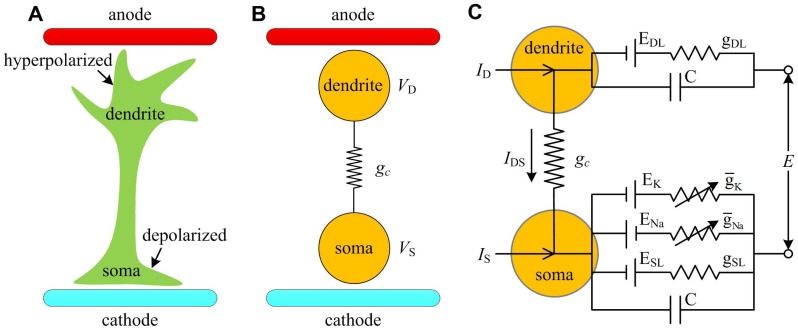
Two-compartment neuron model to extracellular electric fields. (A) A simplified schematic of the spatial polarization in the neuron induced by extracellular electric field. The membrane near the anode is hyperpolarized, which is depolarized near the cathode. (B) To represent this spatial polarization effect, the neuron is replaced by two compartments, which respectively represent soma and dendrite. (C) The currents and conductances for two-compartment neuron. There are Na^+^, K^+^, passive leakage and capacitive currents in somatic compartment, whereas the dendritic compartment only contains a passive leakage current and a capacitive current.

Previous modeling [34]–[36] and experimental [9]–[11], [19], [20] studies have demonstrated that extracellular electric field could induce spatial polarization in the neuron. The membrane potential near anode is hyperpolarized and near cathode is depolarized, which is shown in Figure 1A. The minimal neuronal unit to represent this spatial polarization should at least have two spatially separated compartments [33], [35], [36]. In our previous study [33], we proposed a two-compartment model (Figure 1B and 1C) to describe the interactions between extracellular electric field and neuronal activities. It is a reduced version of the Pinsky-Rinzel model [37], which is commonly used to describe the membrane dynamics of pyramidal neuron under electric field stimulus [35], [36], [38]. Figure 1C shows the currents and conductances for each compartment of the neuron.

The two compartments are soma and dendrite, which are connected by an internal coupling conductance *g_c_*. Their transmembrane potentials are respectively denoted by *V*_S_ and *V*_D_, and the equations to describe their states are
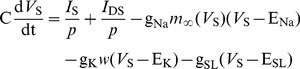
(1)

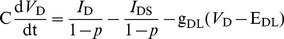
(2)


The five terms on the right side of equation (1) describe the currents on somatic membrane, which are synaptic input current *I*_S_, internal current *I*_DS_ that flows from dendrite to soma, inward Na^+^, outward K^+^ and passive leakage currents, respectively. The three terms on the right side of equation (2) describe the currents on dendritic membrane, which are synaptic input current *I*_D_, internal current *I*_DS_ and passive leakage current. E_Na_ and E_K_ are the reversal potentials of Na^+^ and K^+^ channels on somatic membrane; E_SL_ and E_DL_ are the reversal potentials of leakage channel on somatic and dendritic membrane; 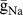, 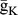, 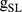 and 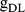 are the corresponding maximum conductances; C is the membrane capacitance; *p* and 1−*p* are geometric parameters that respectively characterize the proportion of area occupied by the soma and dendrite.

In equation (1), *w* is the activation variable for K^+^ current, which represents the probability of activation gate being in the open state for K^+^ channel. It is governed by
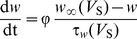
(3)where 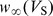 and 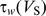 are the steady-state value and time constant for variable *w*. Moreover, 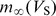 in equation (1) is the steady-state value of the probability of Na^+^ channel being in the open state. 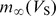, 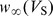 and 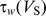 are all functions of somatic membrane potential *V*_S_, which can be described by
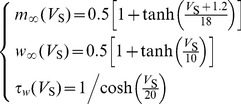
(4)


The orientation of extracellular electric field *E* in our model is parallel to the somatic-dendritic axis, which is in accordance with most experimental studies of individual pyramidal cells to electric fields [18], [20], [39]–[42]. The electric field *E* could induce a polarization between two neuronal compartments and modulate internal current *I*_DS_ [33], [35], [36]. Without electric field *E*, the internal current is 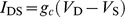. For the neuron in the presence of extracellular electric field, the internal current *I*_DS_ can be expressed as
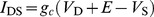
(5)


Since we focus on the spike initiation dynamics of the neuron to extracellular electric fields, the synaptic current *I*_S_ and *I*_D_ are respectively set to 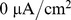 in the following study. The numerical values of the parameters in equations (1)–(5) are shown in Table 1 [33].

**Table 1:** Numerical values for the parameters in the two-compartment model.

Parameters	Values	Parameters	Values
C	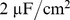	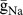	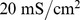
E_Na_	50 mV	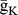	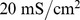
E_K_	−100 mV	g_SL_	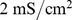
E_SL_	−70 mV	g_DL_	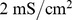
E_DL_	−70 mV	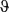	0.15 (unitless)

**Figure 2:**
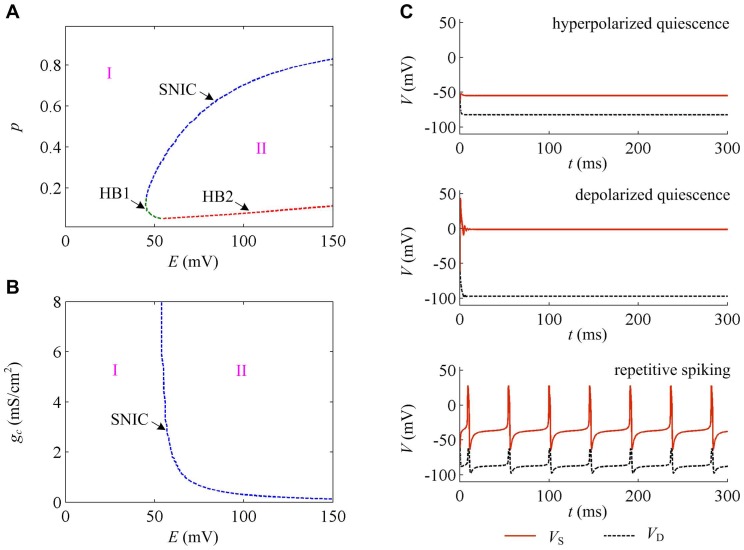
Dynamical properties of the neuron to electric fields. (A) Two-parameter bifurcation diagram in (*p*, *E*) parameter space. (B) Two-parameter bifurcation diagram in (*g_c_*, *E*) parameter space. (C) Different spiking behaviors exhibited by the neuron to electric fields. In (A) and (B), region I indicates quiescent state and region II indicates repetitive spiking state. ‘HB1’ refers to subcritical Hopf bifurcation, ‘HB2’ refers to supercritical Hopf bifurcation, and ‘SNIC’ refers to saddle-node on invariant circle bifurcation.

Through our previous study, it is found that neuron can generate subcritical Hopf bifurcation (HB1), supercritical Hopf bifurcation (HB2) and saddle-node on invariant circle (SNIC) bifurcation as geometric parameter *p* changes, while it can only generate SNIC bifurcation as internal coupling conductance *g_c_* changes [33]. The corresponding two-parameter bifurcation diagrams are shown in Figures 2A and 2B. The bifurcation curve divides the (*p*, *E*) and (*g_c_*, *E*) parameter space into two regions, i.e., region I and II. In region I, neuron is in quiescent state, and in region II it exhibits repetitive spiking behavior (Figure 2C). Moreover, there are two different quiescent states, which are depolarized quiescence and hyperpolarized quiescence (Figure 2C). Since there is only a leakage current in dendritic compartment, it cannot generate spike in the observed range of *E*, which at most exhibits subthreshold oscillation along with *V*_S_ (Figure 2C).

## Results

### Dynamical properties of neuronal spike initiation to extracellular electric field with different geometric parameters

**Figure 3:**
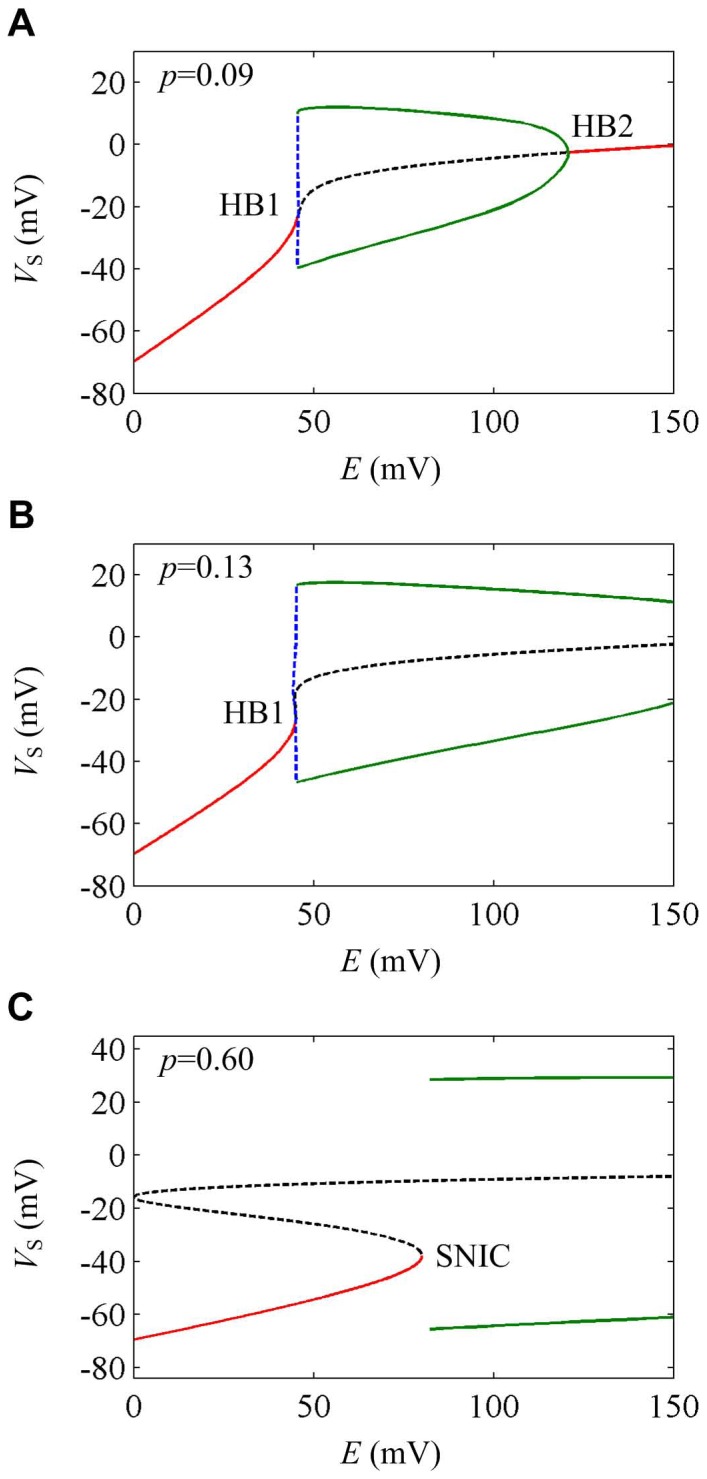
Three types of one-parameter bifurcation by varying geometric parameter. The bifurcation parameter is electric field *E* and the geometric parameter is (A) *p* = 0.09, (B) *p* = 0.13 and (C) *p* = 0.60, respectively. The stable equilibrium is indicated by red solid line and unstable one is black dotted line. The stable limit cycle is indicated by green solid line and unstable one is blue dotted line.

As geometric parameter *p* changes from 0 to 1, the neuron can generate three types of bifurcation to extracellular electric fields. The corresponding sample one-parameter bifurcation diagrams are shown in Figure 3. For 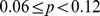, neuron first generates repetitive spiking through a subcritical Hopf bifurcation (HB1), and then cease this repetitive behavior through a supercritical Hopf bifurcation (HB2) when electric field *E* changes from 0 mV to 150 mV (Figure 3A). For 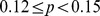, neuron generates repetitive spiking through a subcritical Hopf bifurcation (HB1) and never stops this behavior for 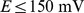 (Figure 3B). However, the neuron generates repetitive spiking through a SNIC bifurcation for 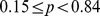 and keeps on spiking when 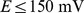 (Figure 3C). When geometric parameter *p* is too high or too low, such as 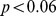 or 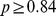, there is no bifurcation occurring for 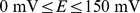. Next, we will use stability and phase plane analysis to further investigate the dynamical properties of the spike initiation in these three cases.

To facilitate stability analysis, we rewrite our two-compartment neuron in the following form
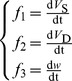
(6)


At the equilibrium points of the neuron, there is
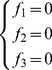
(7)


The Jacobian matrix of the neuron at the equilibrium point has the following form
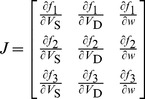
(8)


For 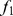, 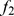 and 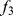, we have
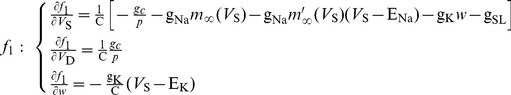
(9)where 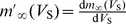.
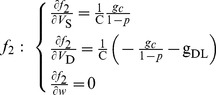
(10)

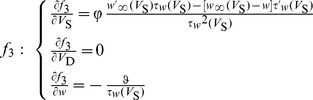
(11)where 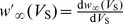 and 
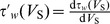
.

From 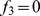 in equation (7), we have 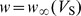 at the equilibrium point. Taking this condition into 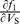 and 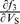, we could obtain the following two equations,
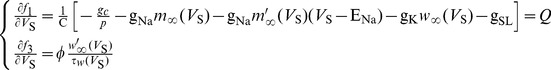
(12)


Substituting equations (9)–(12) into the Jacobian matrix (8), we could obtain
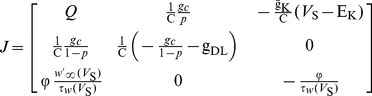
(13)


When *p* = 0.09, neuron generates the left bifurcation (HB1) at 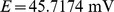. By solving nonlinear equations (7) at this electric field intensity, it is found the equilibrium of the neuron is 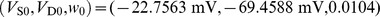. Then, the characteristic polynomial of Jacobian matrix *J* is 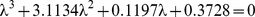. The corresponding characteristic eigenvalues are 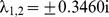 and 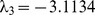. There is a pair of purely imaginary eigenvalues, which corresponds to the non-hyperbolicity condition of Hopf bifurcation [23]. Since there is unstable limit cycle appearing (Figure 3A), the relevant Hopf bifurcation is subcritical. With 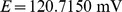, the neuron generates the right bifurcation (HB2). At this electric field intensity, the equilibrium of the neuron is 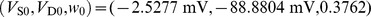, and the characteristic polynomial of Jacobian matrix *J* is 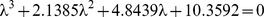. Its corresponding characteristic eigenvalues are 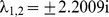 and 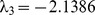. Thus, the right bifurcation is also a Hopf bifurcation [23]. But unlike HB1, there is a stable limit cycle appearing (Figure 3A), so the relevant Hopf bifurcation is supercritical.

**Figure 4:**
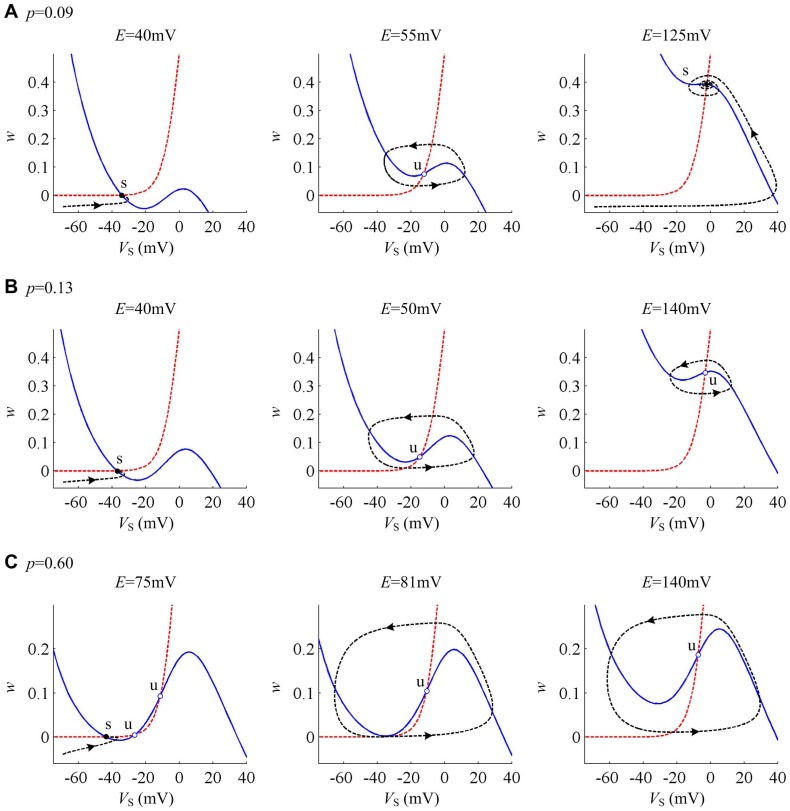
Phase plane analysis about (*w*, *V*_S_) with different geometric parameters. The geometric parameter is (A) *p* = 0.09, (B) *p* = 0.13 and (C) *p* = 0.60, respectively. The blue solid line represents *V*_S_-nullcline and red dotted line is *w*-nullcline. The black dotted line is a sample *V*_S_ trajectory, where arrow indicates the direction of its motion. ‘s’ indicates stable equilibrium and ‘u’ is unstable.

Figure 4A shows the phase plane analysis of the spike initiation dynamics in the case of *p* = 0.09. Since the dendritic compartment only contains a passive leakage current and does not include other ionic currents, the spiking initiation dynamics of the neuron are mainly controlled by the interactions between somatic membrane potential *V*_S_ and its K^+^ channel activation variable *w*. Therefore, although there are three dynamical variables in our model, we could perform phase plane analysis on (*w*, *V*_S_) plane (Figure 4). When 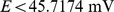, the *V*_S_- and *w*-nullclines interact at a stable fixed point (left panel, Figure 4A). All the *V*_S_-trajectories converge to this stable intersection, so the neuron is at the resting state and there is no repetitive spike generating. The nullcline in phase space for a variable represents the states where the variable neither increases nor decreases [23]. The interaction of two variable nullclines is the equilibrium of the neuron. The increase of electric field *E* has no effects on *w*-nullcline, but could shift the *V*_S_-nullcline to the upper right. Once 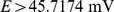, the fixed point is destabilized, and meanwhile there is a stable limit cycle appearing (center panel, Figure 4A). All the *V*_S_-trajectories converge to this stable limit cycle and neuron begins to spike repetitively. The destabilization of the fixed point and the appearance of repetitive spiking in this process are accomplished by subcritical Hopf bifurcation. Then, the *V*_S_-nullcline continues to move to the upper right as *E* further increases. Once 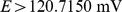, the interaction between *V*_S_- and *w*-nullclines changes to a stable fixed point once more, meanwhile the stable limit cycle disappears (right panel, Figure 4A). In this case, the *V*_S_-trajectories converge to this stable fixed point and repetitive spiking ceases. The stabilization of the fixed point and the qualitative change in neuronal behavior are accomplished by supercritical Hopf bifurcation. Moreover, the stable fixed point is in the suprathreshold range, so the neuron exhibits depolarized quiescent state (center panel, Figure 2C).

When *p* = 0.13, neuron generates bifurcation (HB1) at 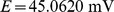. The corresponding characteristic eigenvalues of Jacobian matrix *J* are 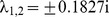 and 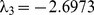. Hence, there is a Hopf bifurcation taking place in the neuron [23]. Since the limit cycle appearing in this process is unstable (Figure 3B), the Hopf bifurcation is subcritical. From the phase portraits in Figure 4B, the interaction between *V*_S_- and *w*-nullclines changes from a stable fixed point to unstable when the bifurcation occurs. Meanwhile, a stable limit cycle appears in the phase portrait which corresponds to repetitive spiking behavior. Unlike the case of *p* = 0.09, the unstable fixed point does not stabilize again and the stable limit cycle always exists for 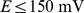. Therefore, the neuron keeps on repetitive spiking in the observed range of *E*.

For *p* = 0.60, neuron generates bifurcation at 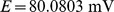. The corresponding characteristic eigenvalues of Jacobian matrix *J* are 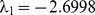, 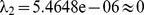 and 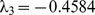. Apparently, this satisfies the non-hyperbolicity condition of the saddle-node bifurcation [23]. From the phase portraits in Figure 4C, it is found there are three interactions between *V*_S_- and *w*-nullclines when 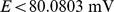. Since the leftmost fixed point is stable and the other two are unstable, neuron exists in quiescent state. As electric field *E* increases, the *V*_S_-nullcline shifts to the upper right and the distance between the left two intersections gets closer. Once 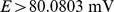, these two intersections coalesce and annihilate each other. Meanwhile, a stable limit cycle appears and neuron begins to spike repetitively. This process corresponds to the SNIC bifurcation. After that, the *V*_S_- and *w*-nullclines always interact at an unstable fixed point and the stable limit cycle always exists in the observed range of *E*. So the neuron always exhibits repetitive spiking for 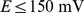.

### Dynamical properties of neuronal spike initiation to extracellular electric field with different internal coupling conductances

**Figure 5:**
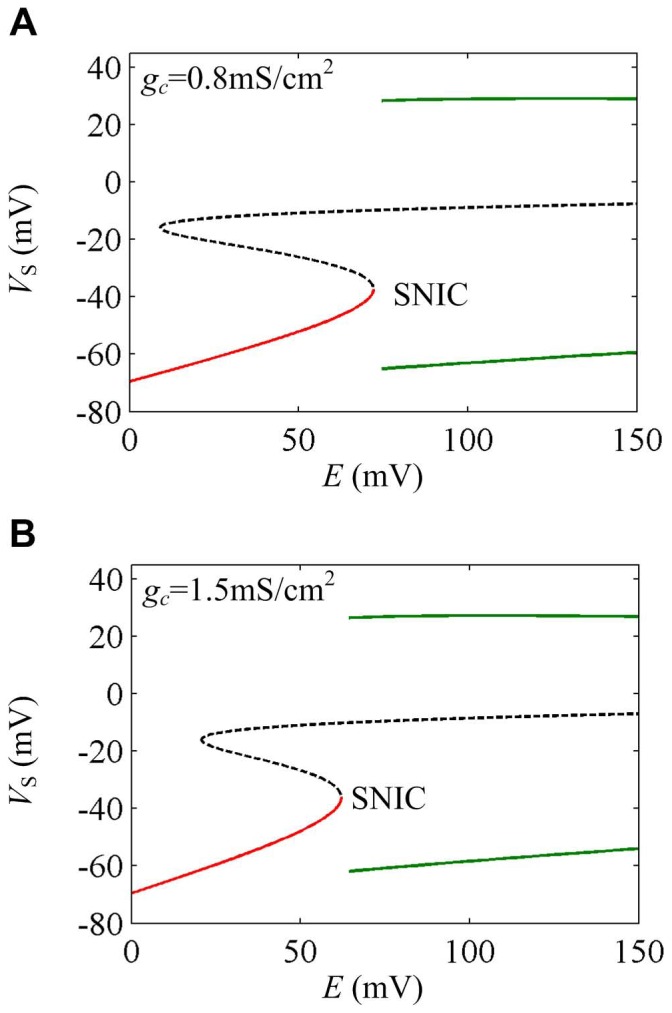
Two sample one-parameter bifurcation diagrams by varying internal coupling conductance. The bifurcation parameter is electric field *E* and the internal coupling conductance is (A) *g_c_* = 0.8 mS/cm^2^ and (B) *g_c_* = 1.5 mS/cm^2^. The stable equilibrium is indicated by red solid line and unstable one is black dotted line. The stable limit cycle is indicated by green solid line and unstable one is blue dotted line.

As internal coupling conductance *g_c_* changes, the neuron can only generate SNIC bifurcation to extracellular electric fields. The corresponding sample one-parameter bifurcation diagrams are shown in Figure 5. That is, the internal coupling conductance *g_c_* can only change the electric field value at the bifurcation point, which cannot alter the bifurcation type.

For 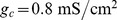, neuron generates bifurcation at 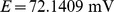. The corresponding characteristic eigenvalues of Jacobian matrix *J* are 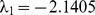, 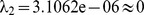 and 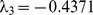. For 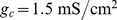, neuron generates bifurcation at 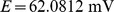. The corresponding characteristic eigenvalues of Jacobian matrix *J* are 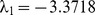, 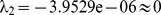 and 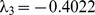. Since each case has a zero characteristic eigenvalue, the corresponding bifurcations are both SNIC bifurcation [23].

**Figure 6:**
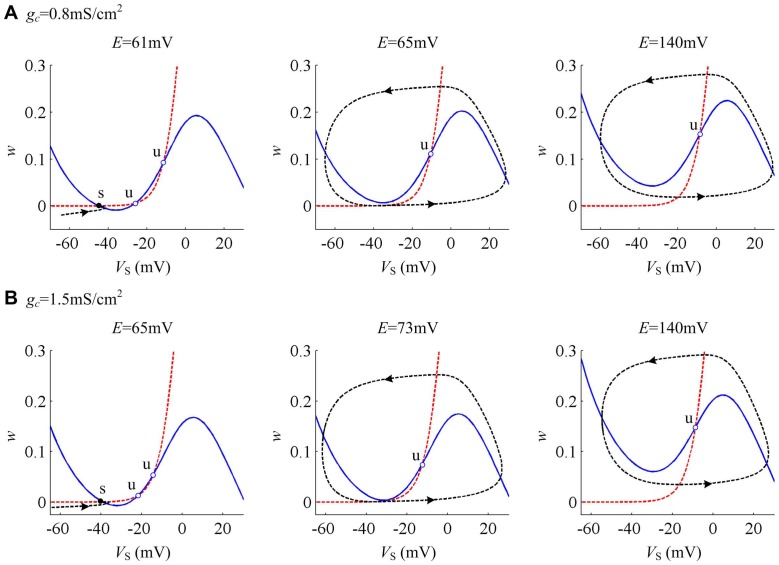
Phase plane analysis about (*w*, *V*_S_) with different internal coupling conductances. The internal coupling conductance is (A) *g_c_* = 0.8 mS/cm^2^ and (B) *g_c_* = 1.5 mS/cm^2^. Blue solid line represents *V*_S_-nullcline and red dotted line is *w*-nullcline. Black dotted line is a sample *V*_S_ trajectory, where arrow indicates its direction of motion. ‘s’ indicates stable equilibrium and ‘u’ is unstable.

Whether 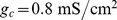 or 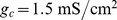, it can be observed that the interactions between *V*_S_- and *w*-nullclines both have three fixed points before bifurcation occurs from the phase portraits in Figure 6. When the bifurcation takes place, the left two fixed points coalesce and annihilate each other. Then, two nullclines interact at only an unstable point. Since there is a stable limit cycle simultaneously appearing, neuron starts to spike repetitively. This process corresponds to the SNIC bifurcation. For 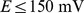, the stable limit cycle always exists in the phase portraits and the neuron always exhibits repetitive spiking.

### Biophysical basis of the spiking initiation dynamics modulated by extracellular electric fields

The neuronal membrane currents with different directions, i.e., inward or outward, could produce different effects on membrane potentials. The inward current mainly leads to the depolarization of membrane potential, while the outward current mainly hyperpolarizes membrane potential [23], [24], [43]. The relative activation properties at the subthreshold potentials between inward and outward currents are tightly related to the dynamical bifurcation of the neuron [24]. When SNIC bifurcation takes place, the outward current is very weak or has not yet been activated at perithreshold potentials, and the activated inward current could drive membrane potential to slowly pass through threshold without competition, which enables neuron to maintain slow repetitive spiking. On the contrary, when Hopf bifurcation occurs, the outward current is relatively strong or has been activated at perithreshold potentials. To generate action potential, the inward current must be activated more rapidly and outrun the outward current to produce suprathreshold depolarization. To summary, a relative increase in outward current encourages the occurrence of Hopf bifurcation, and a relative increase in inward current encourages the occurrence of SNIC bifurcation [24], [43]. Moreover, it is found that the relation between steady-state membrane current *I* and membrane potential *V* (i.e., *I*-*V* curve) in the absence of external stimulus could qualitatively reflect the nonlinear competition between inward and outward currents [23], [24]. Specifically, a Hopf bifurcation corresponds to a monotonic *I*-*V* curve, whereas SNIC bifurcation corresponds to a non-monotonic *I*-*V* curve which has a local maximum value. Thus, to explore the biophysical basis of the spike initiation dynamics modulated by electric fields, we could analyze the activation properties of inward and outward membrane currents at the subthreshold potentials as well as the steady-state *I*-*V* curve properties in the case of different geometric and internal coupling parameters.

Since the dendrite only contains a leakage current, we focus on the relation between somatic steady-state membrane current *I*_SS_ and its membrane potential *V*_S_ (*I*_SS_-*V*_S_ curve). The somatic steady-state membrane current *I*_SS_ is defined as the sum of currents in somatic chamber, which can be written as
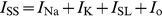
(14)where 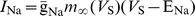 is the steady-state Na^+^ current, 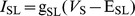 is the leakage current. Since there is 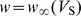 at steady state, the steady-state K^+^ current becomes 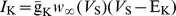. 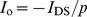 is the steady-state internal current that flows from soma to dendrite and the geometric parameter should be included. In the absence of electric field, i.e., *E* = 0 mV, according to 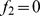 [equation (7)], we have
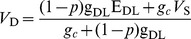
(15)


Then, the steady-state internal current *I*_o_ in equation (14) becomes
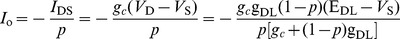
(16)


For somatic chamber, this is an outward current.

**Figure 7:**
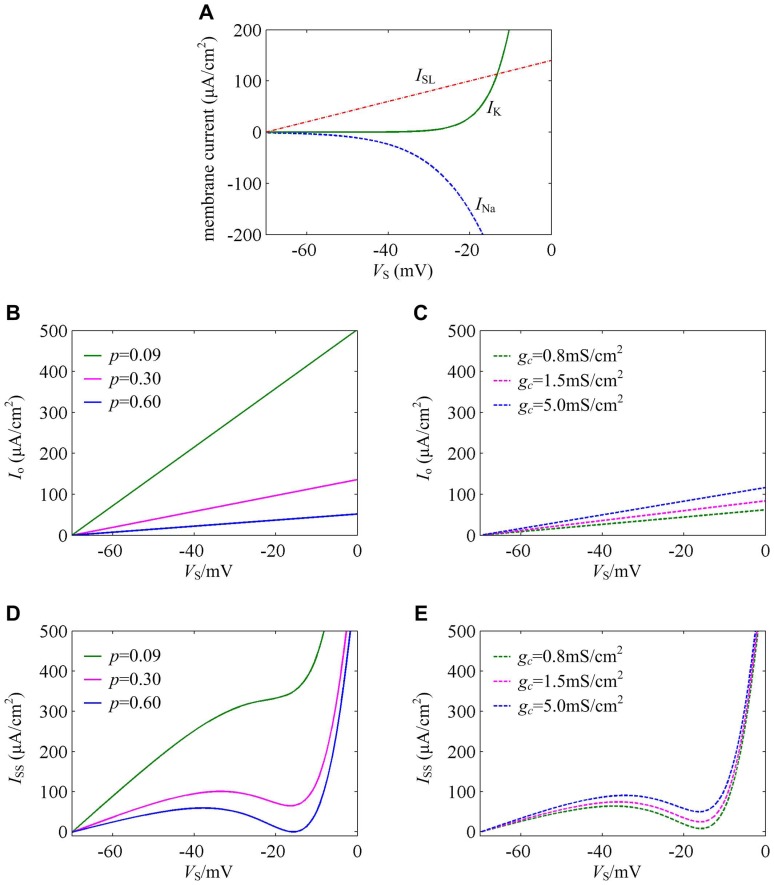
Activation properties of somatic membrane currents at the subthreshold potentials. (A) The relation between somatic *I*_Na_, *I*_K_, *I*_SL_ currents and its membrane potential *V*_S_ at the subthreshold potentials. The activations of these three currents are all not affected by geometric parameter and internal coupling conductance. (B) The relation between the internal current *I*_o_ flowing from soma to dendrite and somatic membrane potential *V*_S_ (*I*_o_-*V*_S_ curve) at the subthreshold potentials with *p* = 0.09, 0.30 and 0.60. (C) *I*_o_-*V*_S_ curves at the subthreshold potentials with *g_c_* = 0.8 mS/cm^2^, 1.5 mS/cm^2^ and 5.0 mS/cm^2^. (D) *I*_SS_-*V*_S_ curves at the subthreshold potentials with *p* = 0.09, 0.30 and 0.60. (E) *I*_SS_-*V*_S_ curves at the subthreshold potentials with *g_c_* = 0.8 mS/cm^2^, 1.5 mS/cm^2^ and 5.0 mS/cm^2^.

From the expressions of steady-state Na^+^ current *I*_Na_, K^+^ current *I*_K_ and leakage current *I*_SL_, it can be found that all of them do not contain geometric parameter *p* and internal coupling conductance *g_c_*. Thus, the change of these two parameters cannot alter the activation properties of *I*_Na_, *I*_K_ and *I*_SL_ at the subthreshold potentials (Figure 7A). From the results in Figure 7A, we can also observe that the inward *I*_Na_ is activated at lower *V*_S_ than outward *I*_K_.

However, both of geometric parameter *p* and internal coupling conductance *g_c_* could affect the activation property of internal current *I*_o_ at subthreshold potentials, which are shown in Figures 7B and 7C. When the geometric parameter *p* is low, the intensity of the internal current *I*_o_ flowing out of soma is very large (*p* = 0.09, Figure 7B). In this case, although inward *I*_Na_ is activated at lower *V*_S_ than outward *I*_K_, the high intensity outward *I*_o_ makes the total outward current become much stronger than inward current at perithreshold potentials, which encourages the occurrence of Hopf bifurcation. From Figure 7D, it can be found the *I*_SS_-*V*_S_ curve in this case is monotonic and does not have local maximum value. Thus, the spike initiation is generated through a Hopf bifurcation (Figure 3A). As geometric parameter *p* increases, the intensity of outward *I*_o_ gets smaller, whereas the activation property of inward current *I*_Na_ remains unchanged, which leads to a relative decrease in outward current. Once *p* exceeds a critical value, the *I*_SS_-*V*_S_ curve changes from monotonic to non-monotonic, which has a local maximum value (*p* = 0.30, Figure 7D). It indicates that the outward current in this case cannot out-compete inward current at perithreshold potentials, and then the membrane potential could be forced to slowly pass through threshold by activated inward *I*_Na_. Thus, the spike initiation of the neuron to electric field is switched from Hopf bifurcation to SNIC bifurcation. As *p* further increases, the intensity of outward *I*_o_ gets even smaller (*p* = 0.60, Figure 7B), which leads to a bigger decrease in outward current relative to inward *I*_Na_. In this case, the *I*_SS_-*V*_S_ curve keeps non-monotonic (*p* = 0.60, Figure 7D), which corresponds to a SNIC bifurcation (Figure 3C).

On the contrary, the intensity of outward current *I*_o_ increases as internal coupling conductance *g_c_* increases (Figure 7C). However, compared with the case of geometric parameter *p*, the increasing range is much smaller, which could not qualitatively alter the nonlinear competition between inward and outward currents. In this case, the *I*_SS_-*V*_S_ curves for different *g_c_* values are always non-monotonic (Figure 7E). Thus, the neuron could only generate SNIC bifurcation to electric fields as *g_c_* changes (Figure 5).

## Conclusions

Based on a reduced two-compartment model, we have studied the dynamical and biophysical mechanism of neuronal spike initiation to extracellular electric fields in this paper. In our previous study [33], it has been shown that this model could generate different bifurcations (i.e., Hopf or SNIC bifurcation) to electric fields as geometric parameter changes, whereas it could only generated SNIC bifurcation as internal coupling conductance changes. However, it still lacks of a complete description of how varying two parameters induce different effects on neuronal spiking initiation dynamics to electric fields.

In this study, we first use stability theory and phase plane analysis to further investigate the dynamical properties of spike initiation induced by these two parameters in detail. It has been shown that geometric parameter variation could lead to different types of characteristic eigenvalues of the Jacobian matrix appearing at the equilibrium point, whereas internal coupling conductance could not. With phase plane analysis, it is shown that varying geometric parameter could alter the mode of interactions between *V*_S_- and *w*-nullclines on the phase portraits, whereas varying another parameter could not. All these results show that the geometric parameter could qualitatively alter the dynamical properties and bifurcation mechanisms of neuronal spike initiation modulated by extracellular electric fields.

Then, we explore the biophysical basis of spike initiation dynamics of the neuron to electric fields. By analyzing the activation properties of inward and outward membrane currents at the subthreshold potentials, it is found that geometric parameter and internal coupling conductance do not affect the activation properties of somatic Na^+^, K^+^ and leakage currents, whereas they could alter the intensity of the internal current flowing from soma to dendrite. Specifically, the internal current intensity greatly decreases as geometric parameter increases, which makes the spike initiation mechanism switch from Hopf bifurcation to SNIC bifurcation. Although the internal current intensity increases as internal coupling conductance increases, the increasing range is much smaller relative to the case of geometric parameter, which could not qualitatively alter the nonlinear competition between inward and outward currents. Then, the spike initiation could occur only through SNIC bifurcation. Hence, it can be concluded that geometric parameter could greatly change the internal current intensity to qualitatively alter the dynamical mechanism of neuronal spike initiation, which further highlight the spatial polarization effects of extracellular electric field. All these findings demonstrate that geometric parameter is a crucial factor in determining neuronal spike initiation dynamics to electric fields. In previous experimental and modeling studies, it has been proposed that morphological feature plays a key role in neuromodulatory effects of electric field. For instance, it could not only determine the electric field threshold for triggering action potentials [8], [9], [18], [20]–[22], but also control the spatial polarization effects induced by electric field [9], [19], [20], [34]. Thus, our results could provide further support for these previous proposals from the spike initiation dynamical point of view.

In addition, our work describes a connection between neuronal biophysical properties and their spike initiation dynamics to electric fields, which is useful to interpret the functional significance of these properties in neural coding. To our knowledge, this is the first study to explore the biophysical basis of spike initiation of the neuron to electric field stimulus. The results could be used to give some general explanations of the corresponding experimental phenomena under electric field stimulus, which in turn could be tested by experimental recording. Moreover, our reduced two-compartment model and modeling approach as well as analysis method could also provide a new perspective to uncover the underlying mechanism of how neuron encodes external electric field stimulus.
